# Anatomical Variations of the Median Nerve: A Cadaveric Study

**DOI:** 10.3390/neurolint14030054

**Published:** 2022-08-23

**Authors:** Manuel Encarnacion, Renat Nurmukhametov, Rossi Evelyn Barrientos, Dmitry Melchenko, Evgeniy Goncharov, Edwin Bernard, Jose Mogorron Huerta, Jean Francois Uhl, Ibrahim E. Efe, Nicola Montemurro, Issael Ramirez

**Affiliations:** 1 Department of Neurosurgery, Russian People’s Friendship University, 121359 Moscow, Russia; 2 Department of Spinal Surgery, Central Clinical Hospital of the Russian Academy of Sciences, 121359 Moscow, Russia; 3 Department of Pathological Anatomy, Central Clinical Hospital of the Russian Academy of Sciences, 121359 Moscow, Russia; 4 Traumatology and Orthopedics Center, Central Clinical Hospital of the Russian Academy of Sciences, 121359 Moscow, Russia; 5 UNESCO Research in Digital Anatomy, University of Paris Rene Descartes, 75006 Paris, France; 6 Department of Neurosurgery, Charité–Universitätsmedizin Berlin, Corporate Member of Freie Universität Berlin, Humboldt-Universität zu Berlin, and Berlin Institute of Health, 10117 Berlin, Germany; 7 Department of Neurosurgery, Azienda Ospedaliera Universitaria Pisana (AOUP), University of Pisa, 56100 Pisa, Italy; 8 Neurosurgery Oncology Fellow Royal Melbourne Hospital, Melbourne, VIC 3000, Australia

**Keywords:** anatomical variations, median nerve, corpses, brachial plexus, peripheral nerves

## Abstract

Objectives: Variations in the morphological anatomy of the median nerve such as formation, distribution, and communication have been well documented. All these variations should be taken into account when practicing any surgical approach for the treatment of injuries affecting the median nerve. Furthermore, they are of the utmost importance for interpretation of the clinical presentation. Methods: The objective of this investigation was to determine the anatomical variations in the formation of the median nerve in cadavers at the Forensic Pathology department in Central Clinical Hospital of the Academy of Sciences of the Russian Federation between January 2022 and April 2022. A descriptive, cross-sectional, and prospective information source study was conducted on 42 anatomical bodies (corpses) and 84 brachial plexuses. Results: After analyzing the results obtained in this investigation, we concluded that the median nerve presented variation in its formation in 22.6% of the investigated cases. These variations were more common in males (81.8%) than females (18.2%). The anatomical variation was unilateral in 7.1% and bilateral in 19% of all anatomical bodies examined. Conclusions: The median nerve presented a great number of variations in its formation in roughly 23% of the anatomical bodies, with male being the predominant gender. Furthermore, the most frequent region of formation was the axillary region (92.9%). For clinicians, it is important to remember these variations during surgical procedures in this area and during brachial plexus block.

## 1. Introduction

The median nerve is one of the terminal branches of the brachial plexus and is formed by two roots, the lateral root from the lateral cord and the medial root from the median cord [1]; however, several studies have shown that variations in its formation and relationships with other anatomical structures can occur [2,3,4]. Median nerve formation by two roots is seen in 48–88.5% of cases [2,3,4]. Formation by three or more roots has been well documented in the literature; however, few papers have reported its incidence. These additional roots generally arise from the lateral cord of the brachial plexus, with variation reported to range from 14.2% to 20%. Rarely, the additional root may arise from the medial cord or the anterior division of the middle trunk of the brachial plexus [5]. In clinical-surgical practice, knowledge of the formation pattern of peripheral nerves, the topographical spaces in which they are found, and the relationships they establish with vascular structures, in addition to their distribution in the different muscle compartments, are of great importance. The median nerve, in its formation and relationships, undergoes several variations as pointed out by Natsis et al. [6]. Neural communication between nerves may lead to confusing clinical and electrodiagnostic findings. Thereby, awareness of the diversity of the communication between median nerve origin and nerves in its vicinity is needed to avoid misdiagnosis of peripheral nerve lesions. The anatomical variations in the median nerve have been highlighted in the context of their clinical importance. Normal limb function may not be disturbed by these variations in cases of regional anesthetic blocks, surgical procedures, axillary dissection in elective surgeries, trauma involving the trunks of the brachial plexus, nerve transpositions, and vascular surgeries [7]. Considering the importance of these anatomical variations in the medial nerve in humans, we investigated and reported our anatomical variation findings in a group of anatomical bodies (corpses). The aim of this anatomical study is to report the incidence, origin, number of anatomical root variation, course, side (unilateral or bilateral), and anatomical relationships of the origin of the formation of the median nerve in anatomical bodies.

## 2. Materials and Methods

### 2.1. Data Acquisition

The study population is represented by 42 anatomical bodies (corpses) of both genders, in equal numbers, analyzed at the Forensic Pathology Department of the Central Clinical Hospital of the Academy of Sciences of the Russian Federation, Moscow, Russia. A descriptive, cross-sectional study was carried out to report a description and measurement of the characteristics observed in the population under study during the time period between January 2022 and April 2022. The inclusion criteria were: (1) age over 18 years old, (2) cadavers with both axillary regions in a fresh condition, (3) no previous dissection work in the axillary regions, (4) no state of decomposition, and (4) corpses that were dissected at the Institute’s headquarters. The exclusion criteria were: (1) age under 18 years old, (2) corpses with infectious diseases, (3) corpses with upper extremity trauma, and (4) corpses with the presence of surgical procedures in the upper extremity.

### 2.2. Dissection Technique

Data collection was carried out through the dissection of 42 brachial plexuses in the corpses of both sexes in equal numbers from the Forensic Pathology Department of the Central Clinical Hospital of the Academy of Sciences of the Russian Federation, under the technique’s dissection of this region. The axillary and anterior region of the arm in both extremities was exposed in order to observe the study characteristics of the median nerve. The data collection began with the purpose of processing this information, which was obtained and collected in a previously prepared and validated form. The data was tabulated and graphed in Microsoft Word and Microsoft Excel, in the process of writing the results, conclusions, and recommendations of this study. The anti-plagiarism detection programs Viper and Plagiarism Checker were used to validate this manuscript.

### 2.3. Ethical and Bioethical Principles

To carry out this research, the principle of confidentiality was preserved in order to protect all the information provided, which was used solely for scientific purposes. Likewise, the norms provided by the institution and other ethical principles such as justice, beneficence, autonomy, and non-maleficence were respected.

## 3. Results

**Figure 1:**
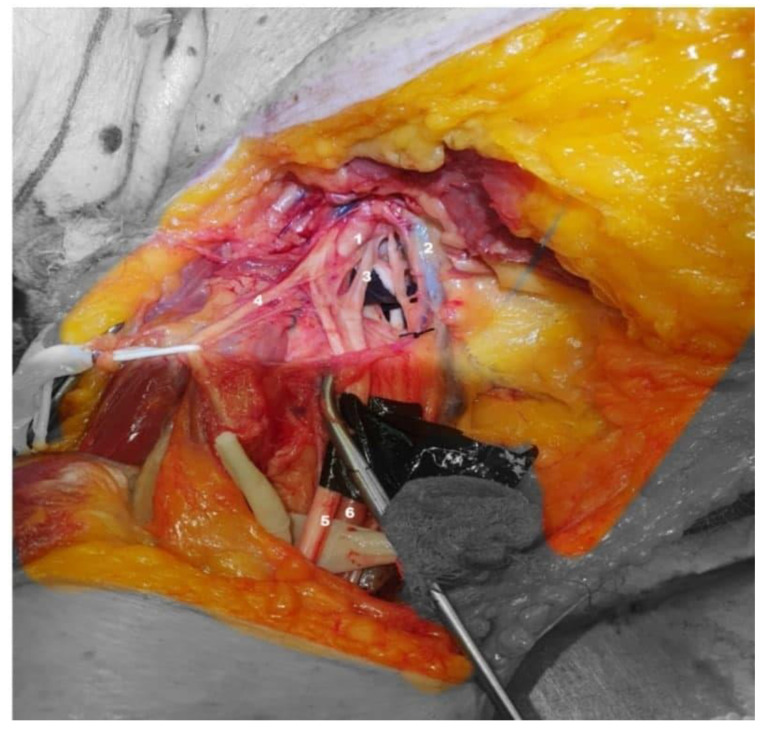
Anterior-inferior view right shoulder: axillary artery (**1**), axillary vein (**2**), medial root to the median nerve (**3**), musculocutaneous nerve (**4**), median nerve (**5**), and axillary artery (**6**).

**Figure 2:**
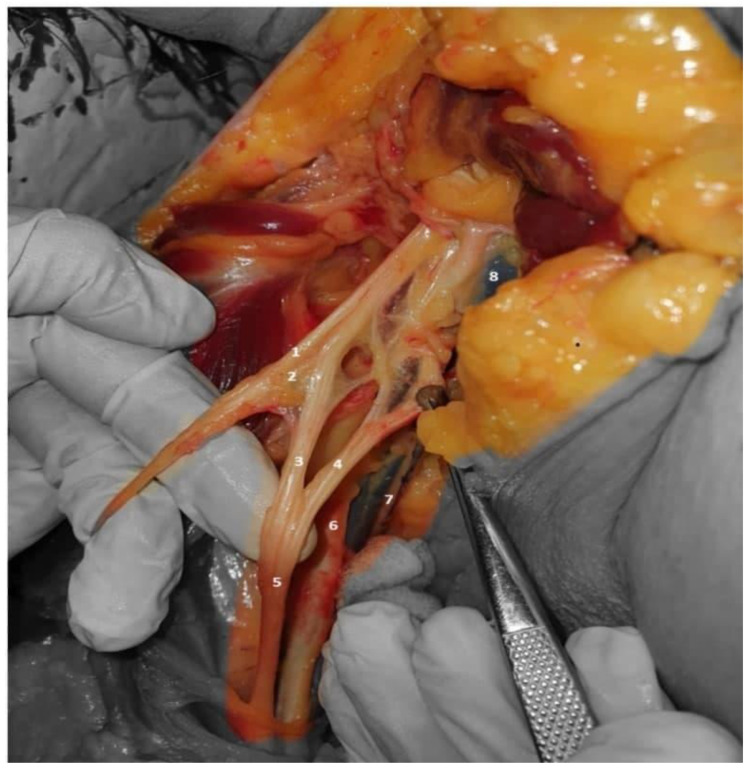
Anterior view, right shoulder: axillary nerve (**1**), musculocutaneous nerves (**2**), lateral and medial roots of the median nerve (**3**,**4**), median nerve (**5**), axillary artery (**6**), thoracodorsal nerve (**7**), and axillary vein (**8**).

**Figure 3:**
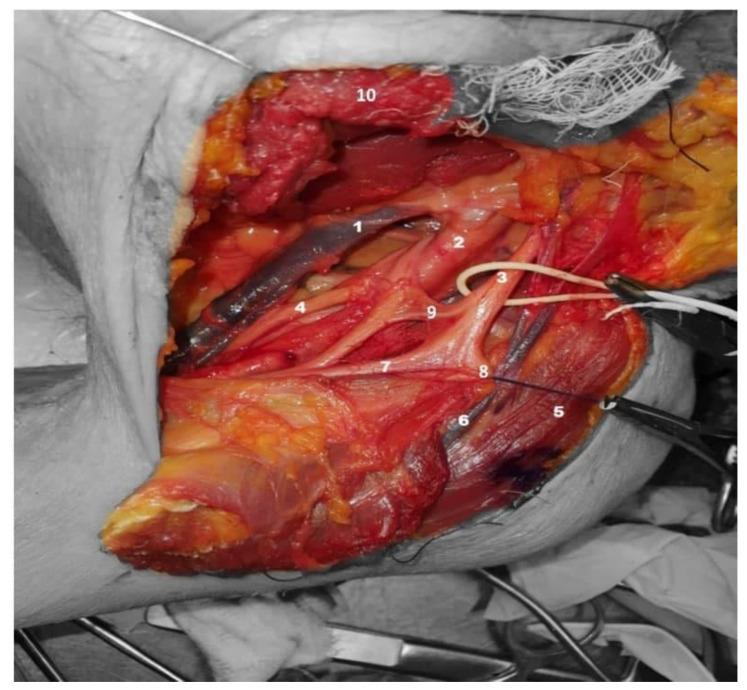
Anterior-superior view, left shoulder: axillary vein (**1**), axillary artery (**2**), lateral cord (**3**), medial cord (**4**), deltoid muscle (**5**), cephalic vein (**6**), lateral root to the median nerve (**7**), musculocutaneous nerve (**8**), anastomotic branch (**9**), and pectoralis minor muscle (**10**).

The origin of the medial nerve and its variations were observed in 42 anatomical bodies (21 male and 21 female) that underwent anatomical dissection to evaluate the anatomical variations in the formation of the median nerve. Of these 42 anatomical bodies, in which both sides were investigated (84 median nerves), 77.4% of all median nerves were formed by 2 branches, representing the usual formation of the median nerve, and only 19 (22.6%) presented a medial nerve origin with more than 2 branches, resulting in an unusual variation. The formation of the plexus originated in the axillary region in 92.9% of cases and in the brachial region in 7.1% of cases. Regarding the distribution by gender, 9 (81.8%) medial nerve origin variations occurred in males and 2 (18.2%) in females. According to the number of branches that form the median nerve, 65 cases of normal origin (with 2 roots) were reported, whereas median nerve origins from 3 branches in 17 (20.2%) specimens and from 4 branches in 2 (2.4%) specimens were found. The origin of the third branch came from the lateral fasciculus in 89.5% of the anatomical variation specimens evaluated and where a fourth branch was found (in 10.5% of all cases of anatomical median nerve variations), it originated from the medial fasciculus. The dissections were performed bilaterally in all the corpses to verify the presence of the variations bilaterally and the results showed that these variations were bilateral in eight anatomical bodies and unilateral in three anatomical bodies. Table 1 and Figure 1, Figure 2 and Figure 3 show all details.

## 4. Discussion

The brachial plexus supplies either motor or sensory innervation to the upper limb. Normally, it is formed by the ventral rami of C5 to C8 and Tl. The plexus arises in the neck and then crosses inferiorly over rib I. Afterwards, it enters the axillary cavity. The median nerve arises, as known, from the plexus brachialis and is normally formed by two roots: a lateral one from the outer cord and a medial one from the inner cord of the plexus brachialis [8]. It receives filaments from the lower three cervical nerves and the first dorsal nerve [8,9]. Of the 84 dissected brachial plexuses, it was found that 22.6% showed an unusual pattern of formation. These data coincide with what Agarwal et al. [2] reported, describing variations in the formation of the median nerve in 24 percent of cases in an unusual way. Variations in the formation of the medial nerve were greater in the male gender (9 cases, 81.8%) while only 18.2 percent corresponded to females. These results are close to those found by Samarawickrama et al. [10], where 60 percent of the corpses were female. In relation to the number of roots, Kumari et al. [11] reported that the median nerve originated from 3 roots in 26.4% and from 4 roots in 1.8% of cases. These data are similar to what we reported in this paper. When analyzing the origin of the additional roots, it was identified that it came from the lateral cord in 20.2% of the anatomical bodies, establishing a significant difference from the data reported by Reis et al. [12], who found that the lateral fascicle originated from an additional root in 8 percent of cases. When analyzing the region of formation of the median nerve, we found that 7.1% formed in the brachial region, data similar to that obtained by Samarawickrama et al. [10], who observed the formation of the nerve in the brachial region, in relation to the brachial artery, in 10%.

Patild et al. [13] pointed out that 20% showed variation in unilateral formation, which is consistent with our results of 27.3% (unilateral) and 72.7% (bilateral). Chitra et al. [14] found 4 abnormalities in 36 cadavers in the formation of the median nerve in the 5 upper extremities. In one of these, the median nerve extremity arose from the individual fused medial and lateral cords of the brachial plexus. In both upper limbs of the same cadaver, three roots (two roots from the lateral cord and one from the medial cord) participated in the formation of the median nerve in 2.7%. In one upper extremity, the median nerve was made up of three roots and the third root was from the musculocutaneous nerve in 1.38 percent. The median nerve was formed by four roots in an upper part; three of them were from the lateral cord and one from the medial cord of the brachial plexus in 1.38%. Similarly, Mat Taib et al. [15] showed that two-rooted median nerve formation accounts for 63.6% and 72.7% for the left and right upper extremity, respectively. Three other variations in median nerve formation: for one root, three roots, and four roots, were also observed. Three-root formation in the median nerve was found in approximately 36.4% in the left upper extremity and 18.2% in the right. More variations were observed in the right upper limb [15]. Variation in the median nerve was observed in a previous paper, such as Bharti et al. [16], in which variation was observed in the morphology of the median nerve, where formation of the median nerve by more than two roots was noted after the dissection of two corpses and the presented variation was unilateral. It should be noted that this type of variation might increase the risk of median nerve injury in surgical operations of the axilla and may lessen the blood supply of the upper extremity by compressing the vessel due to the very close course of the second lateral root of the median nerve to the axillary artery [8]. Chitra et al. [17], during routine dissection of the upper extremity of a male cadaver, found that bilateral variations in the median nerve on the left side the medial root continued to the middle of the arm and joined the lateral root and formed the median nerve in the arm instead of the axilla, whereas on the right side the nerve, it was formed from two lateral roots and one medial. In addition to these anastomoses, others found that anomalous neural connections between the median and ulnar nerves in the upper limb area can aggravate the clinical and anatomical picture: Martin–Gruber anastomosis (MGA), Marinacci anastomosis (MA), Riche–Cannieu anastomosis (RCA), and Berrettini anastomosis (BA). The reported prevalence rates and characteristics of these anastomoses vary significantly between studies [18,19,20,21]. MGA is the most common nerve anastomosis in the upper extremities, and it crosses from the median nerve to the ulnar nerve. Proximal MGA is an under-recognized anastomosis between the ulnar and median nerves at or above the elbow and should not be missed during nerve conduction studies [22]. Rodriguez-Niedenführ et al. [23] reported in their study that the incidence of Martin–Gruber anastomosis was 21.2% and it occurred in 20% of the 55 male cadavers and in 22.2% of the 63 female cadavers. This equal distribution between males and females was not found in our study, where anatomical variations in the formation of the median nerve were more common in males (81.8%) than females (18.2%). It is impossible to establish from this study why there is such a big difference among sexes; however, more embryological studies are needed to understand whether these anatomical variations are under hormonal influence or other epigenetic factors.

Medical accidents caused by surgeons performing procedures without sufficient experience have become a substantial challenge in society [24]. Variations such as as the ones reported are of great significance and have a clinical or surgical impact, as damage in the axillary region may lead to paralysis of the anterior compartment muscles of the arm (biceps brachii, brachialis, and coracobrachialis), motor disability of the elbow joint (flexion), and sensory deficiency in the lateral compartment of the forearm [8]. Although, in recent years, the development of new neurosurgical techniques and 3D devices have helped surgeons to improve their knowledge of surgical anatomy, real laboratory anatomical dissections are needed to safely perform surgeries [24,25,26,27].

### Limitations of the Study

The main limitations of this study are the heterogeneity of the patients (age, chronic pathologies, unknown history of any genetic diseases) and the relatively small sample of corpses. Another limitation is that our anatomical study reflects a single-center experience and, therefore, all dissection were carried out using the same technique, with a very small probability that some anatomical variations were missed. This can create some problems in the quality and risk of bias assessment; however, this bias was minimized as the 42 anatomical bodies that met the inclusion criteria were sequentially analyzed. Additional prospective studies should be conducted in an international, multi-center setting with a large sample size to assess the presence and incidence of these anatomical variations in the median nerve.

## 5. Conclusions

The median nerve presented a great number of variations in its formation in roughly 23% of anatomical bodies, with male being the predominant gender. Furthermore, the most frequent region of formation was the axillary region. The knowledge of anatomical variations regarding hand innervation has a significant importance, particularly when considering physical examination, prognosis, diagnosis, and surgical treatment. If these variations are not valued, mistakes and consequences are inevitable. For surgeons, it is important to remember these variations when surgical procedures are performed in the armpit or during axillary dissection. It is important for hand, orthopedic, and neurosurgeons to be aware of these anatomic variations in order to explain paradoxical motor and sensory loss in patients. Additionally, for anesthesiologists, it is important to be aware of the atypical formation and location of the median nerve, which is critical for anesthetists performing the brachial plexus block.

## Figures and Tables

**Table 1:** Anatomical variations in the formation of the median nerve.

	N°	(%)
Overall anatomical bodies	42	100
Sex		
Male	21/42	50
Female	21/42	50
Area of origin of the median nerve		
Axillary region	78/84	92.9
Brachial region	6/84	7.1
Anatomical variation * in the formation of the median nerve	19/84	22.6
Male	9/42	81.8
Female	2/42	18.2
Number of roots that form the median nerve		
2	65/84	77.4
3	17/84	20.2
4	2/84	2.4
Anatomical variation * of the median nerve according to the side of the body		
Unilateral	3/42	7.1
Bilateral	8/42	19
Origin of the additional variation * root in median nerve formation		
Medial fascicle	2/84	2.4
Lateral fascicle	17/84	20.2

* more than two roots.

## Data Availability

Not applicable.
